# Understanding motor control in health and disease: classic single (*n* = 1) observations

**DOI:** 10.1007/s00221-020-05763-5

**Published:** 2020-03-14

**Authors:** Bastiaan R. Bloem, Mariana H. G. Monje, Jose A. Obeso

**Affiliations:** 1 Department of Neurology, Centre of Expertise for Parkinson and Movement Disorders, Donders Institute for Brain, Cognition and Behaviour, Radboud University Medical Centre, PO Box 9101 (947), 6500 HB Nijmegen, The Netherlands; 2 HM-CINAC, Hospital Universitario HM Puerta del Sur, Universidad CEU-San Pablo, 28938 Móstoles, Madrid Spain; 3 Medical School, CEU-San Pablo University, Madrid, Spain; 4 CIBERNED (Center for Networked Biomedical Research on Neurodegenerative Diseases), Instituto Carlos III, Madrid, Spain

**Keywords:** Case report, Physiology, Motor control, Movement disorders

## Abstract

The field of neuroscience is increasingly dominated by a preferred use of big data, where analysis of large numbers has become an essential area of development. We here draw attention to the importance of smaller numbers, and more specifically, to the historical and continued importance of detailed and judiciously performed studies in single healthy volunteers or single patients with a unique clinical presentation, as an important approach to study normal functions of the nervous system, and to understand the pathophysiology underlying neurological movement disorders. We illustrate this by discussing several historical examples and by summarising Professor John Rothwell’s impressive body of work in single-patient studies, highlighting some of his seminal *n* = 1 studies that have had a great impact on the field. In doing so, we hope to provide a powerful incentive for the next generation of neuroscientists to keep appreciating the value of detailed analyses of single observations.

We live in an era of big data, where using large numbers increasingly matter and have become an important area of development (Beam and Kohane 2018). The field of neuroscience is certainly no exception. This is becoming evident, for example, in the world of predictive modelling for persons living with chronic neurological conditions, where new and sometimes unexpected insights are being derived from hypothesis-free observations made in very large populations, with many observations on a wide range of different variables, often with very long follow-up periods (Fereshtehnejad et al. 2015; Liu et al. 2017; Schrag et al. 2019). At the same time, it remains crucially important to appreciate the unique importance of meticulous and carefully elaborated observations made in much smaller groups of research participants. And these groups can be as small as a single patient or even a single healthy volunteer. In fact, detailed and judiciously performed studies in single patients with a unique clinical presentation represents a classic neurological approach (van der Velden et al. 2018). Indeed, observing particular clinical signs and symptoms in a person with a focal lesion in the nervous system can be understood, under special circumstances, as a “natural experiment” capable of unravelling crucial information about the normal functions of the central nervous system. Just think of the archetypal example of Phineas Gage, the railroad labourer who sustained a remarkable focal brain injury after an explosion that drilled a metal rod straight through his left frontal lobe (Macmillan 2000). The dramatic personality changes that he manifested subsequently highlighted the critical importance of the frontal lobe in regulating important neurological functions such as character and impulse control.

Our own area of research (i.e., the basal ganglia, Parkinson’s disease and other movement disorders) offers plenty of examples. Indeed, there are quite a few of such observations (Table 1) and some were fundamentally important regarding this current article and the neuroscientific contributions of Professor John Rothwell at this time. For example, the single-case observations of Gowers and Ormerod of patients with movement disorders and hepatic cirrhosis guided Kinnier Wilson to recognize his acclaimed observations. Wilson studied the brain of three patients not only to describe the first basal ganglia disease but also to start reasoning about the function of those nuclei at the base of the brain (Wilson 1912). Wilson allegedly was the first neurologist to try to replicate the impact of lesions in the basal ganglia (i.e. the lenticular nucleus) in monkeys, only to be somewhat puzzled and discouraged by the seemingly little impact that ensued. This led to his conclusions: “Whatever functions the corpus striatum once possessed there is no experimental evidence in apes to show that it exercises any motor function comparable to that of the motor cortex. There is no evidence to suggest that it is a centre for so-called automatic movements. It is electrically unexcitable, and comparatively large unilateral lesions do not give rise to any unmistakable motor phenomena” (Wilson 1914).

**Table 1:** Examples of influential single-case (*n* = 1) studies that have greatly contributed to our understanding of the functions of the basal ganglia and that helped to elucidate the pathophysiology of Parkinson’s disease and other movement disorders

Case description	Main findings	Interpretation/relevance	Paper
One patient with ipsilateral involuntary movements following partial recovery from acute hemiparesis. Sensory loss was present	No lesion in the motor tract. Lesion at the ventral third of the contralateral caudate the globus pallidus and the lateral thalamus and subthalamic area	Lack of proprioception may produce similar movements Postural motor activity is modulated by sensory feedback	Hammond (1890)
One case with unilateral parkinsonism	A contralateral tuberculoma within the substantia nigra was found	Antedated the localization of substantia nigra as basis for parkinsonism put forth subsequently	Blocq and Marinescu (1893)
One patient with a haemorrhage in the subthalamic nucleus	Showed hemichorea-hemiballism contralateral to the lesion side	STN exerting inhibition onto basal ganglia output	Martin (1927)
One patient with myoclonic epilepsy starting in his 40s	First patient with features of cortical reflex myoclonus. Cortical somatosensory evoked potentials discovered in humans	The delay between the stimulation and the response and the distribution of the responses show the cortical origin of the discharges. Primitive technology did not prevent a fundamental observation	Dawson (1947)
56-year-old female diabetic patient with dystonia of the left arm of vascular origin	Complete necrosis of the right putamen and head of the caudate nucleus as well as demyelination of the globus pallidus	Focal dystonia can be secondary to focal lesions of the lenticular nucleus	López-Aydillo and Sanz-Ibáñez (1956)
Two PD patients with unintended thalamic lesions, one of whom showed tremor abolition	The ventral thalamus was lesion rather than the globus pallidus. Obliteration of the anterior choroidal artery suggested as treatment of PD tremor	The engagement of the thalamus in PD tremor and potential therapeutic importance for stereotactic surgery was described	Cooper (1953)
70-year-old man with acute severe hemiballism in the left extremities	Four months later, the central portion of the right cerebral peduncle was sectioned. After transient hemiparesis, he recovered and involuntary movements were never present again. Left with hand/fingers clumsiness as only motor deficit	Corticospinal fibres from precentral motor cortex are not essential to maintain power of skeletal musculature. Fine skilful movements were affected	Bucy and Keplinger (1960)
One patient with Parkinson’s disease treated with surgery develops hemiballism	Lesion in the zona incerta, fasciculus thalamicus and ventrolateral thalamic nucleus	Chorea and ballism secondary to a lesion sparing the subthalamic nucleus. Hemiballism suggested as a complex disturbance rather than a single pathological state	Dierssen et al. (1961)
One patient with post-anoxic myoclonic	Treatment with 13 different drugs and thalamotomy 5HTP p.o. improved generalized myoclonus	Beginning of therapeutic of myoclonus	Lhermitte et al. (1971)
62-year-old man with left-side hemiparesis. Self-observation and neuroanatomical considerations after a stroke	Automatic skilled movements were difficult to perform with sufficient strength, speed and coordination	Automatized manual skills can be broken down into a series of particular co-ordinated finger movements. The neuroanatomical localization of the neural program responsible may be organized at the spinal level rather than in the cortex	Brodal (1973)
8-year-old boy with 18-month history of left limb hemi-dystonia	Right lenticular nucleus astrocytoma found in CT brain scan and post-mortem	Symptomatic dystonia secondary to focal lesions in the lenticular nucleus	Narbona et al. (1984)

In 1946, in the incipient clinical research unit at the National Hospital for Nervous Diseases (Queen Square, London), George Dawson studied in detail the first patient ever recognized with cortical myoclonus and discovered cortical somatosensory evoked potentials as a result (Dawson 1947). The brain of that patient was later studied by Greenfield who described cerebellar atrophy, which was decades later realized to be an essential mechanism leading to abnormal cortical excitability in cortical myoclonus (Bhatia et al. 1995).

Paul Bucy, a very astute and active neurosurgeon, wondered about the function of the pyramidal tract. He observed how a patient with hemiballism in whom a pyramidotomy was effectively made (as the only possible therapy to stop the severe life-threatening movements) recovered and was left with minimal motor clumsiness on the affected side (Bucy and Keplinger 1960). The striking global recovery of motor power led Bucy to wonder if the pyramidal tract is needed to sustain normal motor control in humans.

Alf Brodal was a well-known neuroanatomist from Oslo, whose book “Neurological Anatomy in Relation to Clinical Medicine” (published in 1969) was a fundamental reference in the 1970s and 1980s. The book contained several other brilliant accounts; in one of these, he provided an incontestable argument against using the term “extrapyramidal” system and, accordingly, “extrapyramidal” syndrome (chapter 4, pp. 182–183). In 1972, while being a visiting professor at Coimbra University, Brodal himself suffered a left hemisphere stroke with among others right-sided classic hemiparesis. He recovered substantially over the following months but was, somewhat to his own surprise, left with difficulties to carry out fine, skilled manual and finger movements with the right hand, such as tying of a tie, particularly when performed without visual control. Indeed, he lucidly expressed his disability as “his fingers did not know the next move” (Brodal 1973). Clearly, his lenticulo-capsular infarction (no neuroimaging available then) had disturbed the motor circuit and the automatic (habitual) system.

In the late 1990s, Bill Langston’s recognition of a single patient (six other similar patients were later identified as well) who subacutely developed severe and irreversible parkinsonism after receiving repeated intravenous injections of 1-methyl-4-phenyl-1,2,3,6-tetrahydropyridine (MPTP), a synthetic variant of heroin, raised firm awareness to the possible role played by environmental neurotoxins in the origin of Parkinson’s disease (Langston et al. 1983). The role of environmental factors in the aetiopathogenesis of PD is still a major area of research (Dorsey et al. 2018). Furthermore, the observation triggered hectic “in vitro” studies to understand the mechanism of action of the toxin and led to a now classic animal model of nigrostriatal neurodegeneration (Burns et al. 1983).

Observations on single patients can also impact on treatments. For example, the striking benefit of thalamotomy against parkinsonian tremor was derived from incidental observations on an initial patient operated by Irving Cooper in whom the thalamus was accidentally lesioned secondary to anterior choroidal artery occlusion (Cooper 1953). The anti-parkinsonian effect of amantadine was serendipitously discovered when the drug was prescribed to a shaking Parkinson’s patient to treat the flu, which was at the time its original therapeutic indication (Schwab et al. 1969). In the current era, it suffices to mention two examples with personal involvement of some of us. One is a patient with typical orthostatic tremor to whom JAO indicated that had she not responded to drug treatments, surgery was always an option since “all tremors respond to thalamic surgery” (sic). Clearly, not a cautious assertion! Two years later, the lady returned ready for surgery. Nothing had been done before for orthostatic tremor and of course, there was no tremor-related activity to record from the Vim as the patient laid down in the operating room. Nevertheless, we decided to place the electrodes in the Vim (recognized by the typical proprioceptive responses) and the patient exhibited a remarkable benefit of deep brain stimulation (Guridi et al. 2008). Several other patients have benefited afterwards. Similarly, it is now appreciated that cycling can be remarkably preserved in patients with Parkinson’s disease, even among those who are completely grounded because of freezing of gait. This notion came from an initial observation in a single patient (Snijders and Bloem 2010), which was followed by observations in larger series of patients (Snijders et al. 2012; Alberts et al. 2011), and eventually culminated in the application of cycling on a stationary bicycle as an elegant way of delivering an aerobic intervention into the patients’ own homes (van der Kolk et al. 2019).

Accordingly, seemingly anecdotal observations such as these, when correctly understood and ascertained, do have clinical and neuroscientific value and emphasise the importance of single-case observations, as a catalyst for new insights and a source of inspiration for future research. As Jan Vandenbroucke elegantly pointed out in a thought-provoking commentary, single-case observations are all but a lack of evidence, but instead represent a particular type of evidence that can motivate and inform further research efforts (Vandenbroucke 1999).

Here, we put forward and discuss the importance of carefully elaborated single-case observations to understand the human nervous system and to throw light onto disease mechanisms. This has been particularly relevant for motor control and the human nervous system. We dedicate this article to Professor John Rothwell, who deserves particular credit for recognising the tremendous potential of such approach over decades of research on the pathophysiology of motor control and movement disorders (Table 2).

**Table 2:** Examples of single-case studies by Professor John Rothwell and their implications

Case description	Methodology	Main findings	Interpretation/relevance	Paper
20-year-old man with history of jerks of the right forearm while writing	Forced supination by torque motor Focal anaesthesia	Active pronation produced beats of pronation/supination tremor Forced supination elicited a burst of tremor Anaesthesia temporarily abolished difficulty in movement and tremor	“Writing tremor” caused by proprioceptive critical stimulus. Insights into mechanisms of focal task-specific dystonia	Rothwell et al. (1979)
36-year-old man deafferented by a severe peripheral sensory neuropathy of unknown cause	EEG Surface EMG Manipulating speed and load of finger movements	No sense of touch and inability to maintain a constant motor output Visual check constantly required for the simplest motor tasks Normal pre- and post-movement EEG potentials Normal bi/triphasic pattern of muscle activation during fast limb movements	In the absence of feedback, the accuracy of a motor programme is a function of its duration There is a post-movement potential in the absence of peripheral feedback, that is potentially mainly related to some central re-efferent copy mechanism	Rothwell et al. (1982)
20-year-old man with stimulus-sensitive cortical myoclonus and focal epilepsy secondary to focal parietal lesion	Direct cortical recording EEG Long-latency stretch and cutaneous reflexes	Spike (positive–negative) activity revealed by EEG back-averaging preceding the onset of EMG activity in spontaneous jerks Jerks evoked by somatosensory stimuli elicited similar responses Both jerks disappeared after the cortical region resection	Epilepsia partial continua and the cortical reflex myoclonus share common neurophysiological similarities, caused by spontaneous discharges from the same cortical region	Cowan et al. (1986)
55-year-old man with orthostatic tremor	Surface EMG Accelerometer	Tremor of 16 Hz in the legs with unique precipitating factors (standing, not present with movement) Synchronization of tremor burst in equivalent muscles of both legs Similar tremor record in the arms	Orthostatic tremor as an abnormality in the delivery of “feed-forward” postural signals for standing, due to spontaneous oscillations in central control of posture structures	Thompson et al. (1986)
67-year-old woman with acute onset of unilateral upper limb tremor with variability and distractibility, initially suspected of functional origin	EMG frequency analysis Accelerometer MRI	Each tremor pulse corresponds to a short EMG burst Frequency analysis revealed the same phase relationship between burst manifest in all conditions in different muscles Not entrainment Lesion in the contralateral putamen in the MRI	Coherence analysis looking for entrainment of tremor by voluntary movement as a critical tool for the differential diagnosis of organic from functional tremor	McAuley et al. (1998)
26-year-old man with parkinsonism developed due to acquired isolated bilateral lesions of the globus pallidus	Reaction time motor tests Finger dexterity tasks Movement-related cortical potentials Contingent negative variation MRI	Most of the different measures of motor performance were similar to those reported in patients with PD	Output of the lesioned globus pallidus become “pathological” due to the acquired lesion Pallidotomy may “normalized” the output in patients with PD	Kuoppamaki et al. (2005)
53-year-old man with Parkinson's disease surgical lesions of left subthalamic and left globus pallidus nuclei	Reaction time motor tests TMS fMRI Temporal discrimination experiments	Subthalamotomy treatment improved cardinal motor features but lead to hemiballism effectively treated with pallidotomy Sequence learning in motor tasks was absent High percentage of no-go trials compare to go trials lead to a smaller reaction time in the right hand	Beneficial clinical effect of surgery in PD Basal ganglia outflow can be disrupted without an apparent deficit, but its input to the cortex is needed under some novel circumstances to learn move and act normally	Obeso et al. (2009)
49-year-old man with DYT1 dystonia	EMG Playing piano with and without auditory feedback	Paradoxical improvement of generalized dystonia with activity (playing the piano) with and without auditory feedback	Motor programs for some voluntary activities remain preserved in generalized dystonia and thus performing highly skilled and well-consolidated motor activities overcome abnormal motor output due to basal ganglia dysfunction	Kojovic et al. (2012)

Note that this is a selection of exemplary publications that illustrate the scope of approaches used in the various studies and also the range of implications for care and science derived from them

Remarkably, these case descriptions have had very versatile implications. Given John’s background as Professor of Neurophysiology, it is obvious that much of the case descriptions impacted directly on our insights into the physiology of the human motor system, such as the role of afferent sensory information in shaping motor performance (Rothwell et al. 1982), the contribution of the supplementary motor area (SMA) to motor programming (Dick et al. 1986), the involvement of the globus pallidus in normal motor control and the pathophysiology of Parkinson’s disease (Kuoppamaki et al. 2005) or the physiology of corticospinal connections (Di Lazzaro et al. 1996). Invariably, the publications contained very detailed clinical descriptions of the patients involved and some of these have actually helped to define the clinical phenotype, for example that of writer’s cramp (Rothwell et al. 1979). Other publications had clear diagnostic implications, such as the detailed polymyography studies of a patient with a suspected functional tremor that eventually turned out to be caused by a structural underlying lesion (McAuley et al. 1998); the methodology used still helps to distinguish functional from organic tremor today. Still other studies helped to clarify the pathophysiology underlying the various types of movement disorders, some of which had until that time been poorly recognised and certainly insufficiently studied. Examples include detailed work on dystonia (Rothwell et al. 1979; Berardelli et al. 1986), various forms of myoclonus (Cowan et al. 1986; Brown et al. 1991; Di Lazzaro et al. 1996; Marsden et al. 2000), chorea (Di Lazzaro et al. 2006), orthostatic tremor (Thompson et al. 1986), hemimasticatory spasm (Mir et al. 2006) and the Tullio phenomenon (Colebatch et al. 1994). Interestingly, even though John is not an M.D. and therefore, not involved in immediate patient care himself, other case studies provided relevant new insights into treatment options for patients with movement disorders. Examples here include detailed studies into the mechanism of action underlying treatments such as lorazepam (Di Lazzaro et al. 2000), botulinum toxin (Mir et al. 2006) or brain surgery for Parkinson’s disease (Obeso et al. 2009). A particularly attractive study—performed in a dystonic patient while playing the piano with and without auditory feedback—provided provocative new insights into the role of music as novel treatment for persons with dystonia (and perhaps other movement disorders) (Kojovic et al. 2012), an approach that had since gained a lot of momentum among both patients, scientists and clinicians (Bloem et al. 2018).

Taken together, John’s impressive body of work should provide a powerful incentive for the next generation of neuroscientists to keep appreciating the value of detailed analyses of single patients, to study the functioning of the human nervous system in health and disease. John’s publications also highlight how such single-case studies can have an impact on patient care as an incidental but desirable catch, by elucidating the mechanisms underlying not only the efficacy but also the adverse effects of invasive treatments. In fact, one could argue that the advent of personalised precision medicine—with its intrinsic call for tailor-made treatments adjusted to the specific needs and profiles of each individual patient (Dzau et al. 2015)—should only lead to a further rebirth of carefully executed single-case studies. This notion is indeed increasingly recognised when it comes to development of personalised treatments, with the advent of new methodologies such as multiple *n* = 1 trial designs (Schork 2015; Stunnenberg et al. 2018). Single-case studies are therefore not just of historical importance, as we have addressed here, but also continue have great value for the neuroscience of the future.

We like to finish by reminding readers of John’s seminal work on “chronostasis”: the irresistible notion experienced by many when a saccadic eye movement is made to a ticking clock, making the observer think that the second hand takes longer than normal to move to its next position (Yarrow et al. 2001). We have no doubt that this study too was originally triggered by a single-case observation, most likely John sharply observing his own clock—yet another powerful illustration how such anecdotal findings and everyday curiosity can help to advance the field of neuroscience.

## References

[CR1] Alberts JL, Linder SM, Penko AL, Lowe MJ, Phillips M (2011). It’s not about the bike, it’s about the pedaling. Exerc Sport Sci Rev.

[CR2] Beam AL, Kohane IS (2018). Big data and machine learning in health care. JAMA.

[CR3] Berardelli A, Thompson PD, Day BL, Rothwell JC, O'Brien MD, Marsden CD (1986). Dystonia of the legs induced by walking or passive movement of the big toe in a patient with cerebellar ectopia and syringomyelia. Neurology.

[CR4] Bhatia KP, Brown P, Gregory R (1995). Progressive myoclonic ataxia associated with coeliac disease. The myoclonus is of cortical origin, but the pathology is in the cerebellum. Brain.

[CR5] Bloem BR, Pfeijffer IL, Krack P (2018). Art for better health and wellbeing. BMJ.

[CR6] Brodal A (1973). Self-observations and neuro-anatomical considerations after a stroke. Brain.

[CR7] Brown P, Thompson PD, Rothwell JC, Day BL, Marsden CD (1991). A case of postanoxic encephalopathy with cortical action and brainstem reticular reflex myoclonus. Mov Disord.

[CR8] Bucy PC, Keplinger JE (1960). Section of the cerebral peduncles. Trans Am Neurol Assoc.

[CR9] Burns RS, Chiueh CC, Markey SP, Ebert MH, Jacobowitz DM, Kopin IJ (1983). A primate model of parkinsonism: selective destruction of dopaminergic neurons in the pars compacta of the substantia nigra by *N*-methyl-4-phenyl-1,2,3,6-tetrahydropyridine. Proc Natl Acad Sci USA.

[CR10] Colebatch JG, Rothwell JC, Bronstein A, Ludman H (1994). Click-evoked vestibular activation in the Tullio phenomenon. J Neurol Neurosurg Psychiatry.

[CR11] Cooper IS (1953). Anterior chorodial artery ligation for involuntary movements. Science.

[CR12] Cowan JM, Rothwell JC, Wise RJ, Marsden CD (1986). Electrophysiological and positron emission studies in a patient with cortical myoclonus, epilepsia partialis continua and motor epilepsy. J Neurol Neurosurg Psychiatry.

[CR13] Dawson GD (1947). Investigations on a patient subject to myoclonic seizures after sensory stimulation. J Neurol Neurosurg Psychiatry.

[CR14] Di Lazzaro V, Restuccia D, Nardone R (1996). Changes in spinal cord excitability in a patient with rhythmic segmental myoclonus. J Neurol Neurosurg Psychiatry.

[CR15] Di Lazzaro V, Oliviero A, Meglio M, Cioni B, Tamburrini G, Tonali P, Rothwell JC (2000). Direct demonstration of the effect of lorazepam on the excitability of the human motor cortex. Clin Neurophysiol.

[CR16] Di Lazzaro V, Dileone M, Pilato F (2006). Repetitive transcranial magnetic stimulation of the motor cortex for hemichorea. J Neurol Neurosurg Psychiatry.

[CR17] Dick JP, Benecke R, Rothwell JC, Day BL, Marsden CD (1986). Simple and complex movements in a patient with infarction of the right supplementary motor area. Mov Disord.

[CR18] Dierssen G, Bergmann L, Gioino G, Cooper I (1961). Hemiballism following surgery for Parkinson’s disease. Arch Neurol.

[CR19] Dorsey ER, Sherer T, Okun MS, Bloem BR (2018). The emerging evidence of the Parkinson pandemic. J Parkinsons Dis.

[CR20] Dzau VJ, Ginsburg GS, Van Nuys K, Agus D, Goldman D (2015). Aligning incentives to fulfil the promise of personalised medicine. Lancet.

[CR21] Fereshtehnejad SM, Romenets SR, Anang JBM, Latreille V, Gagnon JF, Postuma RB (2015). New clinical subtypes of Parkinson disease and their longitudinal progression a prospective cohort comparison with other phenotypes. JAMA Neurol.

[CR22] Guridi J, Rodriguez-Oroz MC, Arbizu J (2008). Successful thalamic deep brain stimulation for orthostatic tremor. Mov Disord.

[CR23] Hammond G (1890). Original case of athetosis. J Nerv Ment Dis.

[CR24] Kojovic M, Parees I, Sadnicka A (2012). The brighter side of music in dystonia. Arch Neurol.

[CR25] Kuoppamaki M, Rothwell JC, Brown RG, Quinn NP, Bhatia KP, Jahanshahi M (2005). Parkinsonism following bilateral lesions of the globus pallidus: performance on a variety of motor tasks shows similarities with Parkinson's disease. J Neurol Neurosurg Psychiatry.

[CR26] Langston JW, Ballard JW, Tetrud JW, Irwin I (1983). Chronic parkinsonism in humans due to a product of meperidine-analog synthesis. Science.

[CR27] Lhermitte F, Peterfalvi M, Marteau R, Gazengel J, Serdaru M (1971). Analyse pharmacologique d'un cas de myoclonies d’intention et d’action post-anoxiques. Rev Neurol.

[CR28] Liu G, Locascio JJ, Corvol JC, Boot B, Liao Z, Page K, Franco D, Burke K, Jansen IE, Trisini-Lipsanopoulos A, Winder-Rhodes S, Tanner CM, Lang AE, Eberly S, Elbaz A, Brice A, Mangone G, Ravina B, Shoulson I, Cormier-Dequaire F, Heutink P, van Hilten JJ, Barker RA, Williams-Gray CH, Marinus J, Scherzer CR (2017). Prediction of cognition in Parkinson’s disease with a clinical–genetic score: a longitudinal analysis of nine cohorts. Lancet Neurol.

[CR29] López-Aydillo N, Sanz-Ibañez I (1956). A propósito de un caso de distonía de torsión (variante miostática o paralítica) en una diabética glucosúrica. Trabajos Inst Caja Inv BIol.

[CR30] Macmillan M (2000). Restoring Phineas Gage: a 150th retrospective. J Hist Neurosci.

[CR31] Blocq P, Marinescu G (1893). Sur un cas de tremblement Parkinsonien hémiplégique symptomatique d’une tumeur du pédoncule cérébral. Mem Société Biol.

[CR32] Marsden JF, Ashby P, Rothwell JC, Brown P (2000). Phase relationships between cortical and muscle oscillations in cortical myoclonus: electrocorticographic assessment in a single case. Clin Neurophysiol.

[CR33] Martin JP (1927). Hemichorea resulting from a local lesion of the brain. The syndrome of the body of Luys. Brain.

[CR34] McAuley JH, Rothwell JC, Marsden CD, Findley LJ (1998). Electrophysiological aids in distinguishing organic from psychogenic tremor. Neurology.

[CR35] Mir P, Gilio F, Edwards M, Inghilleri M, Bhatia KP, Rothwell JC, Quinn N (2006). Alteration of central motor excitability in a patient with hemimasticatory spasm after treatment with botulinum toxin injections. Mov Disord.

[CR36] Narbona J, Obeso JA, Tuñon T, Martinez-Lage JM, Marsden CD (1984). Hemi-dystonia secondary to localised basal ganglia tumour. J Neurol Neurosurg Psychiatry.

[CR37] Obeso JA, Jahanshahi M, Alvarez L (2009). What can man do without basal ganglia motor output? The effect of combined unilateral subthalamotomy and pallidotomy in a patient with Parkinson's disease. Exp Neurol.

[CR38] Rothwell JC, Traub MM, Marsden CD (1979). Primary writing tremor. J Neurol Neurosurg Psychiatry.

[CR39] Rothwell JC, Traub MM, Day BL, Obeso JA, Thomas PK, Marsden CD (1982). Manual motor performance in a deafferented man. Brain.

[CR40] Schork NJ (2015). Personalized medicine: time for one-person trials. Nature.

[CR41] Schrag A, Anastasiou Z, Ambler G, Noyce A, Walters K (2019). Predicting diagnosis of Parkinson’s disease: a risk algorithm based on primary care presentations. Mov Disord.

[CR42] Schwab RS, England AC, Poskanzer DC, Young RR (1969). Amantadine in the treatment of Parkinson's disease. JAMA.

[CR43] Snijders AH, Bloem BR (2010). Cycling for freezing of gait. N Engl J Med.

[CR44] Snijders AH, van Kesteren M, Bloem BR (2012). Cycling is less affected than walking in freezers of gait. J Neurol Neurosurg Psychiatry.

[CR45] Stunnenberg BC, Raaphorst J, Groenewoud HM (2018). Effect of mexiletine on muscle stiffness in patients with nondystrophic myotonia evaluated using aggregated N-of-1 trials. JAMA.

[CR46] Thompson PD, Rothwell JC, Day BL, Berardelli A, Dick JP, Kachi T, Marsden CD (1986). The physiology of orthostatic tremor. Arch Neurol.

[CR47] van der Kolk NM, de Vries NM, Kessels RPC, Joosten H, Zwinderman AH, Post B, Bloem BR (2019). Effectiveness of home-based and remotely supervised aerobic exercise in Parkinson's disease: a double-blind, randomised controlled trial. Lancet Neurol.

[CR48] van der Velden RMJ, Mulders AEP, Drukker M, Kuijf ML, Leentjens AFG (2018). Network analysis of symptoms in a Parkinson patient using experience sampling data: an n = 1 study. Mov Disord.

[CR49] Vandenbroucke JP (1999). Case reports in an evidence-based world. J R Soc Med.

[CR50] Wilson SAK (1912). Progressive lenticular degeneration: a familial nervous disease associated with cirrhosis of the liver. Brain.

[CR51] Wilson SAK (1914). An experimental research into the anatomy and physiology of the corpus striatum. Brain.

[CR52] Yarrow K, Haggard P, Heal R, Brown P, Rothwell JC (2001). Illusory perceptions of space and time preserve cross-saccadic perceptual continuity. Nature.

